# Treatment of Hailey-Hailey disease with the Janus kinase inhibitor abrocitinib: A case report

**DOI:** 10.1177/2050313X251350332

**Published:** 2025-06-20

**Authors:** Molly Gunyon, Megha Udupa, Farhan Mahmood, William D. Foulkes, Kevin Pehr, Elena Netchiporouk

**Affiliations:** 1 Faculty of Medicine and Health Sciences, McGill University, Montreal, QC, Canada; 2 Division of Dermatology, Department of Medicine, McGill University Health Centre, Montreal, QC, Canada; 3 Division of Experimental Medicine, Department of Medicine, McGill University, Montreal, QC, Canada; 4 Cancer Research Program, Research Institute of the McGill University Health Centre, Montreal, QC, Canada; 5 Department of Human Genetics, McGill University, Montreal, QC, Canada; 6 Cancer Axis, Lady Davis Institute, Jewish General Hospital, Montreal, QC, Canada; 7 Division of Dermatology, Department of Medicine, Jewish General Hospital, McGill University, Montreal, QC, Canada; 8 Lady Davis Institute for Medical Research, Montreal, QC, Canada

**Keywords:** Hailey-Hailey, abrocitinib, JAK inhibitor, genodermatoses

## Abstract

Hailey-Hailey disease is a rare, chronic, autosomal dominant skin disorder characterized by recurrent painful erosions and macerated plaques, primarily affecting intertriginous areas. It is caused by mutations in the *ATP2C1* gene, leading to impaired calcium homeostasis and keratinocyte adhesion. Many patients experience poor disease control despite conventional therapies. We report a case of a female in her 60s with refractory Hailey-Hailey disease affecting the perianal, inguinal, and cervical folds, with painful, eroded plaques resistant to conventional treatments. Despite multiple failed therapies, including methotrexate, dapsone, acitretin, and naltrexone, she showed rapid improvement within 2 weeks of abrocitinib (100 mg daily), a JAK1 inhibitor, with sustained control at 2 months follow-up. JAK inhibitors, initially approved for inflammatory diseases such as atopic dermatitis, are emerging as promising therapies for genodermatoses. By suppressing IL-4/IL-13-driven inflammation, JAK1 inhibition may restore epithelial integrity and reduce chronic skin inflammation. This case adds to growing evidence that JAK inhibitors, particularly abrocitinib, may serve as an effective targeted therapy for refractory Hailey-Hailey disease. Further clinical trials are needed to confirm its long-term efficacy and safety.

## Introduction

Hailey-Hailey disease (HHD), also known as familial benign chronic pemphigus, is a rare autosomal dominant disorder caused by pathogenic mutations in the *ATP2C1* gene, which encodes the human secretory pathway Ca(2+)-ATPase (hSPCA1).^1^ This calcium pump plays a crucial role in maintaining intracellular calcium balance, and its dysfunction leads to keratinocyte adhesion defects, resulting in recurrent painful erosions, macerated plaques, and secondary infections primarily in intertriginous areas such as the axillae, groin, and perianal region.^2^ Histologically, HHD is characterized by suprabasal acantholysis with a “dilapidated brick wall” appearance.^2^ Although treatment focuses on controlling exacerbations, many patients experience persistent symptoms despite long-term use of topical corticosteroids, systemic retinoids, immunosuppressants, and antibiotics, often due to inadequate efficacy or intolerable side effects. Herein, we describe a case of recalcitrant HHD that achieved rapid and sustained remission following treatment with abrocitinib, adding to the emerging evidence supporting the role of JAK inhibition in the management of this challenging genodermatosis.

## Case report

A female in her 60s, Fitzpatrick skin type III, was assessed in our multidisciplinary genodermatology clinic for the management of HHD. She was diagnosed in her twenties following a pathognomonic skin biopsy. Over time, her disease progression shifted from predominant axillary involvement to primarily affecting the inguinal, vulvar, and perianal regions, leading to significant impairment in her quality of life. In October 2023, upon initial assessment, she presented with painful, poorly healing erosions in the folds of her neck, as well as the inguinal and perianal regions. There was no evidence of nail or mucosal involvement. A strong familial history of HHD was noted, with six maternal family members affected across four generations, though her son remained unaffected. Genetic testing (Invitae Corporation, San Francisco, CA, USA) confirmed a pathogenic loss-of-function variant (c.163C>T, p.Arg55*) in the *ATP2C1* gene.

**Figure 1.**
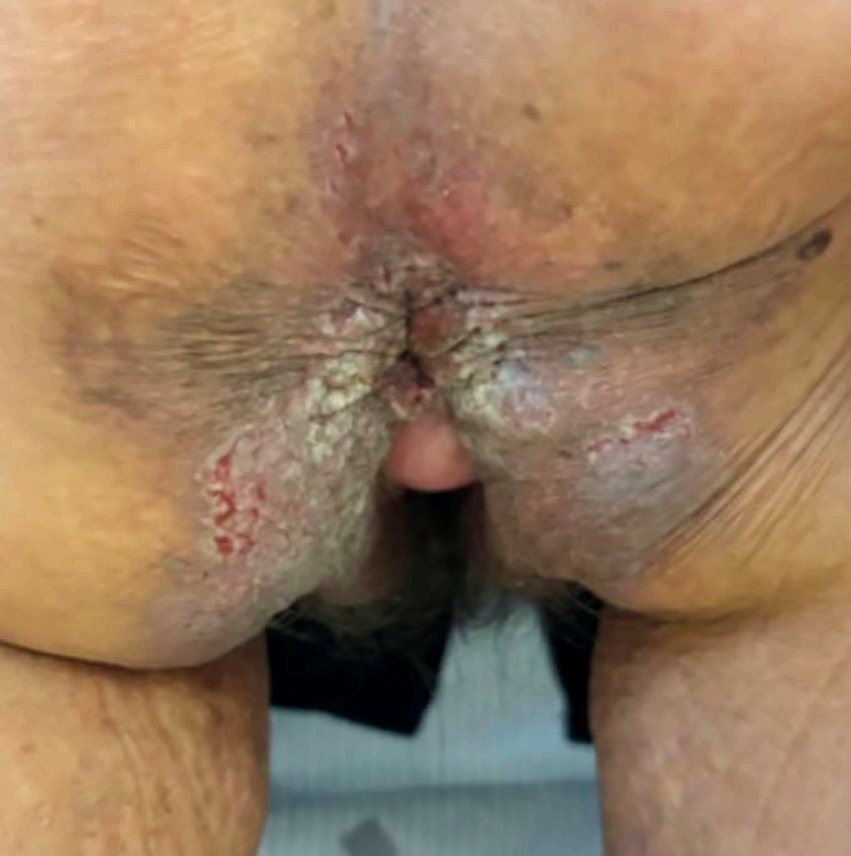
Severe Hailey-Hailey disease prior to abrocitinib treatment. Clinical presentation prior to initiating treatment with abrocitinib. The image demonstrates painful, poorly healing erosions, fissures, and maceration localized to the perianal and adjacent inguinal regions. Significant erythema and lichenification are also noted, indicative of chronic inflammation and recurrent disease exacerbations. The severity of the lesions led to significant impairment in the patient’s quality of life.

**Figure 2.**
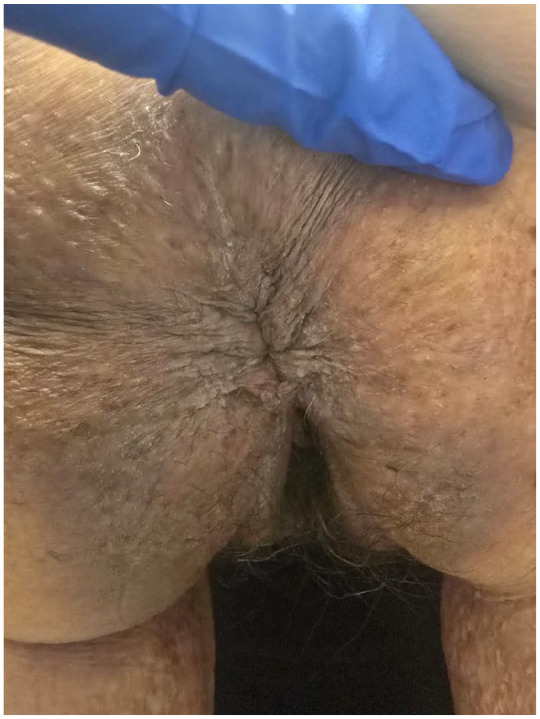
HHD 2 weeks post-abrocitinib treatment. Marked clinical improvement in the perianal region of a female patient with HHD 2 weeks after initiating abrocitinib. Previously observed erosions, fissures, and maceration have resolved, with re-epithelialization and restoration of skin integrity. Minimal postinflammatory hyperpigmentation and lichenification remains, and no active lesions are visible, reflecting sustained disease control. The patient reported significant symptom relief and improved quality of life. HHD: Hailey-Hailey disease.

The patient had undergone multiple therapeutic trials over the years. Oral methotrexate in combination with topical corticosteroids was used for ~15 years but was eventually discontinued due to persistent flare-ups and new lesion development. A subsequent trial of dapsone lasted only 1 month, as it was discontinued due to anemia. Acitretin was then initiated but was stopped after 1 month due to intolerable nausea. At the time of our first assessment, she still had active erosions, prompting the initiation of low-dose naltrexone (5 mg once daily) with concurrent oral magnesium chloride 300 mg once daily. After 5 months, no improvement was observed. Consequently, we applied for off-label public coverage of dupilumab and initiated a trial of topical roflumilast cream 0.3% once daily. However, roflumilast caused significant nausea and was discontinued. Ultimately, the request for dupilumab coverage was declined. Consequently, we secured compassionate access to oral abrocitinib 100 mg once daily. Figure 1 illustrated perianal involvement in November 2024 (pre-abrocitinib). The patient noted improvement within 2 days of initiating abrocitinib and achieved complete resolution of skin lesions within 2 weeks. However, after running out of samples and missing 2 weeks of treatment, her lesions recurred. Upon resuming abrocitinib, she again experienced complete control within 2 weeks. By February 2025, she had maintained complete control of HHD (Figure 2). Reported side effects were limited to mild, tolerable nausea, and constipation.

## Discussion

JAK inhibitors, such as abrocitinib, modulate the JAK–STAT signaling pathway, which plays a key role in inflammation and immune regulation.^3^ Their effectiveness in treating inflammatory skin disorders like atopic dermatitis has led to interest in their application for other chronic dermatoses, including HHD.^3^ The pathogenic loss-of-function mutation in *ATP2C1* observed in HHD results in disrupted calcium homeostasis and impaired keratinocyte adhesion.^4^ This dysfunction contributes to epidermal barrier defects, which further exacerbate inflammation.^4^ Recent studies suggest that IL-4 and IL-13 pathways stimulate eotaxin-3, which inhibits free intracellular calcium release and actin polymerization.^4^ As both processes are crucial for keratinocyte adhesion, type 2 inhibition by dupilumab is hypothesized to restore hSPCA1 function by mitigating inflammation-driven barrier dysfunction and stabilizing actin organization.^4^

Recent evidence supports the efficacy of dupilumab, an IL-4, and IL-13 inhibitor, in treating HHD.^4^ Multiple case reports and small case series have demonstrated its ability to reduce inflammation and improve lesion healing in patients with recalcitrant disease.^4^ To date, there have been a single case report of abrocitinib,^5^ upadacitinib,^6^ and tofacitinib^7^ monotherapy in HHD. In these cases, systemic JAK inhibitors demonstrated rapid resolution of lesions, with sustained control in refractory cases. Reported side effects have generally been mild, with occasional gastrointestinal symptoms such as nausea. Additionally, one report described the use of topical ruxolitinib in conjunction with dupilumab for refractory HHD, suggesting that topical JAK inhibition may serve as an alternative to systemic therapy in localized disease.^8^ However, larger studies are needed to confirm their efficacy and long-term safety.

Our case adds to the growing evidence supporting type 2 inhibitors as a promising therapeutic option for recalcitrant HHD. The rapid and sustained response observed in our patient suggests that JAK inhibition can provide meaningful relief for individuals with severe, treatment-resistant disease. Further research should explore both systemic and topical JAK inhibitors as targeted interventions for HHD and evaluate their long-term safety and efficacy in this rare genodermatosis.
